# Complete remission of renal cell carcinoma with lung carcinomatous lymphangiosis after primary therapy with immune checkpoint inhibitors followed by partial nephrectomy for surgical consolidation

**DOI:** 10.1002/iju5.12427

**Published:** 2022-03-25

**Authors:** Takanari Kambe, Toshinari Yamasaki, Yuta Mine, Hiroki Hagimoto, Hidetoshi Kokubun, Masashi Kubota, Naofumi Tsutsumi, Koji Inoue, Shigeo Hara, Mutsushi Kawakita

**Affiliations:** ^1^ 26330 Department of Urology Kobe City Medical Center General Hospital Kobe Japan; ^2^ Department of Urology Kyoto University Hospital Kyoto Japan; ^3^ 13612 Department of Urology Kurashiki Central Hospital Kurashiki Japan; ^4^ 26330 Department of Pathology Kobe City Medical Center General Hospital Kobe Japan

**Keywords:** carcinomatous lymphangiosis, cytoreductive nephrectomy, immune checkpoint inhibitor, metastatic renal cell carcinoma, robot‐assisted partial nephrectomy

## Abstract

**Introduction:**

Cytoreductive nephrectomy has been used in combination with systemic therapy for the treatment of metastatic renal cell carcinoma, although its efficacy in the era of immune checkpoint inhibitors remains controversial.

**Case presentation:**

A 57‐year‐old woman was diagnosed with left renal cell carcinoma and lung carcinomatous lymphangiosis (cT3aN0M1). After receiving combined immunotherapy, she achieved complete response for the lung metastases and partial response for the primary tumor. After five months of systemic therapy, she underwent partial nephrectomy to remove the primary tumor, followed by eight courses of nivolumab monotherapy. One year postoperatively, she remained recurrence‐free.

**Conclusion:**

Cytoreductive partial nephrectomy for surgical consolidation may be a treatment option for metastatic renal cell carcinoma.

**Abbreviations & Acronyms**
CN: cytoreductive nephrectomy
CPN: cytoreductive partial nephrectomy
CR: complete response
CT: computed tomography
ICI: immune checkpoint inhibitor
mRCC: metastatic renal cell carcinoma
OS: overall survival
PR: partial response
RAPN: robot‐assisted partial nephrectomy
TKI: tyrosine kinase inhibitor
TT: targeted therapy

**Keynote message**

Although there is no consensus regarding the indications, timing, and methods for cytoreductive nephrectomy as part of the multidisciplinary treatment of metastatic renal cell carcinoma, cytoreductive partial nephrectomy after systemic immunotherapy is worth considering in selected patients.

## Introduction

The efficacy of CN in the treatment of mRCC has been discussed. Despite the advent of TKI and ICI therapy, the efficacy and the timing of CN, especially that of CPN, remains controversial. Indeed, there are few retrospective reports comparing CN with CPN combined with systemic therapy. Herein, we report a case of mRCC with lung metastasis, which achieved CR after primary ICI therapy and subsequent RAPN.

## Case presentation

**Fig. 1:**
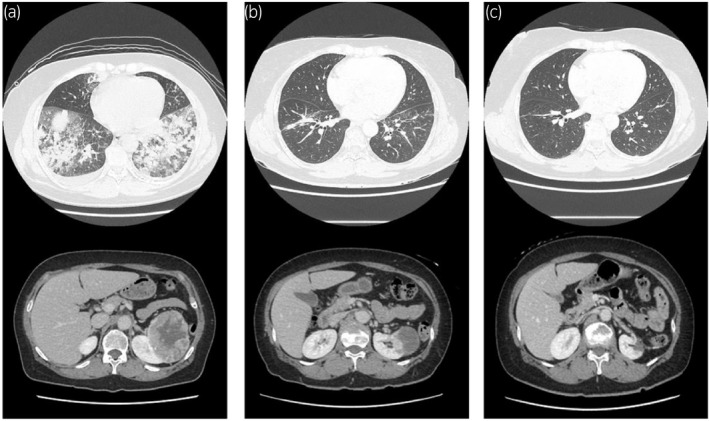
CT images. (a) At the time of initial presentation, bilateral infiltrative shadows can be seen in both lungs and a 85 × 83 mm mass with contrast enhancement at the upper pole of the left kidney. (b) After ICI treatment before surgery, showing CR for the metastasis in the lung and PR for the renal tumor. (c) One year after CPN. There is no local or distal recurrence.

A 57‐year‐old woman with a history of diabetes mellitus and dyslipidemia visited a clinic, complaining a cough that had persisted for a month. Chest radiograph showed bilateral infiltrative shadows, and she was referred to our hospital. Blood tests showed a mildly elevated C‐reactive protein of 2.61 mg/dL, but no other abnormalities were noted. Transthoracic echocardiography showed normal cardiac wall motion. Chest CT showed multiple diffuse patchy infiltrative shadows in the bilateral lung fields (Fig. 1a); she was hospitalized with suspected idiopathic organizing pneumonia. Additionally, a left kidney tumor approximately 90 mm in diameter was incidentally identified by the same CT scan, and she was referred to the department of urology.

**Fig. 2:**
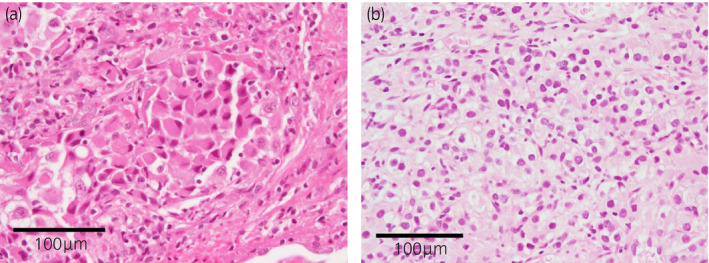
Histopathological images of hematoxylin and eosin staining. (a) Transbronchial lung biopsy shows rhabdoid atypical cells. (b) Percutaneous renal tumor biopsy shows clear cell carcinoma.

**Fig. 3:**
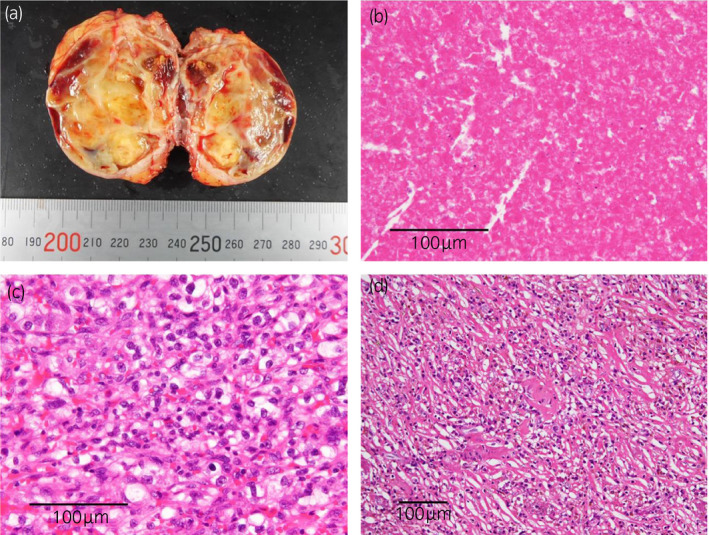
Pathological evaluation of the surgical specimens. (a) Macroscopic image. (b) Coagulative necrotic tissue at the tumor site. (c) An 8 × 6 mm residual tumor. (d) Prominent inflammatory cell infiltration in the tubular region surrounding the tumor.

Methylprednisolone 80 mg/day was started, but no improvement in lung shadow was observed. Dynamic CT showed an 85 × 83 mm enhanced mass at the upper pole of left kidney (Fig. 1a), and RCC was suspected. A transbronchial lung biopsy showed many atypical rhabdoid cells (Fig. 2a), and malignancy was suspected. Additional immunostaining revealed CK AE1/3 positive, CD68 negative, CD10 focal positive, and PAX8 positive tissue, diagnosed as RCC metastasis. Percutaneous renal tumor biopsy revealed high‐grade clear cell RCC (Fig. 2b); thus, we made a diagnosis of stage cT3aN0M1 left RCC and lung metastasis complicated by carcinomatous lymphangiosis, with IMDC intermediate risk. She was started on ipilimumab and nivolumab combined systemic therapy. After the introduction of first course, her respiratory condition temporarily worsened and she was admitted to the Intensive Care Unit; however, her condition gradually improved. Four courses of combination therapy followed by seven courses of nivolumab monotherapy were administered. After achieving CR for lung metastasis and PR for primary tumor, which shrunk to 54 mm in diameter (Fig. 1b), she underwent RAPN for the primary tumor. Surgery was performed using a transperitoneal approach. Renal parenchyma around the tumor was whitish and inflammatory, and the tumor was resected using enucleoresection, preserving as much normal tissue as possible. Inner suture or renorrhaphy were not used, and hemostasis was achieved by soft coagulation with a ball electrode and application of Tachosil^®^ to the resection surface as previously reported.^1^ No perioperative complications were observed. Histopathological findings showed a residual 8 × 6 mm viable cancer cells of Fuhrman grade 3 in the mostly necrotic tumor, with marked inflammatory cell infiltration in the surrounding renal parenchyma (Fig. 3). Renal function was unchanged, with pre‐ and postoperative estimated glomerular filtration rate of 63 and 60, respectively. After eight courses of postoperative nivolumab monotherapy, complete remission was maintained, and systemic therapy was terminated. One year postoperatively, CT scan showed no recurrence (Fig. 1c).

## Discussion

With the advent of new drug therapies, such as ICIs, and surgical methods, such as robotic surgery, the efficacy of CN, especially CPN, needs to be examined.

CN has been reported to prolong OS when combined with interferon α compared to interferon α alone for the treatment of mRCC.^2^, ^3^ Several retrospective studies have shown that the combination of TT and CN prolonged OS.^4^ A subsequent prospective study reported the non‐inferiority of sunitinib alone to sunitinib plus CN, making the combination of TT and CN less meaningful, particularly in patients with poor‐risk mRCC.^5^ Another prospective study compared immediate and delayed CN, and showed that delayed CN prolonged OS.^6^

Recently, several systemic therapies for mRCC have been developed, such as ICI combinations or ICI/TKI combinations.^7^, ^8^, ^9^, ^10^ Since there is no evidence for superiority in them for the first line therapy, it is necessary to decide which regimen to use for each patient. In addition, pulmonary carcinomatous lymphangiosis due to mRCC is rare and, to our knowledge, there have been no reports of the effectiveness of ICI treatment for this clinicopathological entity. There is a report showing a rapid therapeutic effect of nivolumab on carcinomatous lymphangiosis due to lung cancer.^11^ On the other hand, other reports showed transient exacerbations of imaging and respiratory symptoms, which is considered to be pseudoprogression after ICI introduction, as in our case, and it requires caution in treatment.^12^

The role of CN combined with ICI in mRCC has only been reported in a retrospective study where the CN prolonged OS.^13^ Regarding the safety and perioperative complications of CN after ICI, dissection in the appropriate plane may be difficult due to adhesions and inflammatory changes.^14^ However, Singla *et al*., reported no technical problems with the procedure and the perioperative complications were acceptable, and they concluded that CN after ICI was safe and technically feasible.^15^

Primary lesions are sometimes difficult to resect at diagnosis. It was reported that 8.3% of patients were down‐staged in the primary site after prior ICI.^13^ Moreover, there have been several reports where ICI resulted in tumor shrinkage, allowing for radical tumor resection, suggesting that neoadjuvant ICI may enable resection of otherwise unresectable tumors.^16^

CN has frequently been applied for mRCC, however, with the rise of robotic surgery, CPN could also be considered. Although CPN is associated with more concerns regarding oncologic outcomes than CN, a retrospective study comparing CN and CPN in patients with mRCC reported prolonged OS with CPN when the primary tumor was <4 cm.^17^ Several other retrospective studies have reported the benefit of CPN.^18^, ^19^ Furthermore, CPN is expected to preserve renal function, reduce cardiovascular events, and allow for future therapeutic intervention. In the present case, we performed CPN albeit the primary tumor was more than 4 cm even after ICI treatment. This is because the viable lesion appeared to be much smaller than the mass image and CPN was technically feasible. Moreover, we aimed to maintain as much renal function as possible to keep more drug treatment options in case of recurrence. We considered intraoperative careful dissection of the tumor due to peritumoral inflammatory changes, and there was minimal bleeding, no major complications occurred, and renal function was preserved.

Further studies are warranted on the efficacy of the combination of ICIs and CN, the surgical difficulties and frequency of perioperative complications of CN after ICI, and the efficacy of CPN instead of CN.

## Conclusion

We performed RAPN as a curative therapy after prior ICI combinations for mRCC with lung carcinomatous lymphangiosis. ICIs did not significantly impact the difficulty of surgery or perioperative complications. The patient remained in CR 1 year postoperatively.

## Author Contributions

Takanari Kambe: Resources; Supervision; Validation; Visualization; Writing – original draft; Writing – review & editing. Toshinari Yamasaki: Supervision; Validation; Writing – original draft; Writing – review & editing. Yuta Mine: Data curation; Resources. Hiroki Hagimoto: Data curation; Resources. Hidetoshi Kokubun: Data curation; Resources. Masashi Kubota: Data curation; Resources. Naofumi Tsutsumi: Data curation; Resources. Koji Inoue: Data curation; Resources. Shigeo Hara: Data curation; Resources; Visualization. Mutsushi Kawakita: Supervision; Visualization; Writing – original draft; Writing – review & editing.

## Conflict of interest

The authors declare no conflict of interest.

## Approval of the research protocol by an Institutional reviewer board

Not applicable.

## Informed consent

All human subjects provided written informed consent with guarantees of confidentiality.

## Registry and the registration no. of the study/trial

Not applicable.
